# Modification of plant cell walls with hydroxycinnamic acids by BAHD acyltransferases

**DOI:** 10.3389/fpls.2022.1088879

**Published:** 2023-01-17

**Authors:** Niharika Nonavinakere Chandrakanth, Chengcheng Zhang, Jackie Freeman, Wagner Rodrigo de Souza, Laura E. Bartley, Rowan A.C. Mitchell

**Affiliations:** ^1^ Institute of Biological Chemistry, Washington State University, Pullman, WA, United States; ^2^ Department of Microbiology and Plant Biology, University of Oklahoma, Norman, OK, United States; ^3^ Plant Sciences, Rothamsted Research, West Common, Harpenden, Hertfordshire, United Kingdom; ^4^ Center for Natural and Human Sciences, Federal University of ABC, Santo André, Brazil

**Keywords:** ferulic acid, para-coumaric acid, grasses, cell wall, xylan, lignin, plant biotechnology, bioenergy

## Abstract

In the last decade it has become clear that enzymes in the “BAHD” family of acyl-CoA transferases play important roles in the addition of phenolic acids to form ester-linked moieties on cell wall polymers. We focus here on the addition of two such phenolics—the hydroxycinnamates, ferulate and *p*-coumarate—to two cell wall polymers, glucuronoarabinoxylan and to lignin. The resulting ester-linked feruloyl and p-coumaroyl moities are key features of the cell walls of grasses and other commelinid monocots. The capacity of ferulate to participate in radical oxidative coupling means that its addition to glucuronoarabinoxylan or to lignin has profound implications for the properties of the cell wall – allowing respectively oxidative crosslinking to glucuronoarabinoxylan chains or introducing ester bonds into lignin polymers. A subclade of ~10 BAHD genes in grasses is now known to (1) contain genes strongly implicated in addition of *p*-coumarate or ferulate to glucuronoarabinoxylan (2) encode enzymes that add *p*-coumarate or ferulate to lignin precursors. Here, we review the evidence for functions of these genes and the biotechnological applications of manipulating them, discuss our understanding of mechanisms involved, and highlight outstanding questions for future research.

## Background - importance of cell wall hydroxycinnamates

**Figure 1:**
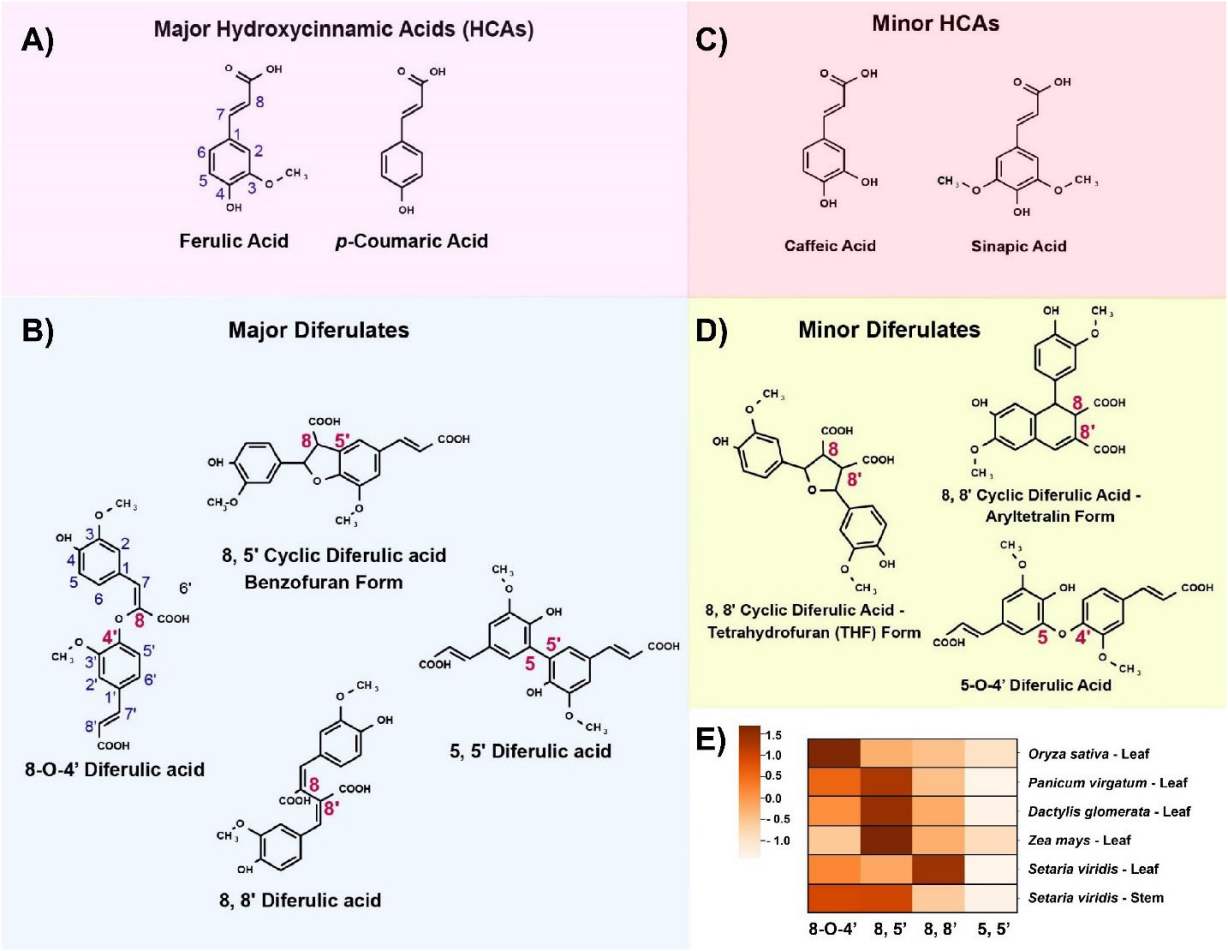
Hydroxycinnamic acid and diferulic acid structures and relative abundances. **(A-D)** Chemical structures of major and minor hydroxycinnamates and diferulates observed from grass cell walls. **(E)** Heatmap represents the relative abundances of major diferulates in *Oryza sativa* leaf tissue (Bartley et al., 2013), *Panicum virgatum* leaf, *Dactylis glomerata* leaf, *Zea mays* leaf (Hatfield et al., 1999), and *Setaria viridis* leaf and stem (de Souza et al., 2018). Relative abundances are Z-scores [(observed value–mean for a given species) /std deviation for that species)].

Cell walls are integral to plant growth and development, encapsulating most cells, dictating their shape and comprising most plant biomass. Cell wall polymer composition and modifications vary across cell types and developmental stages defining the properties of the wall. Primary cell walls are deposited at the cell plate and around expanding cells, and during development must allow for breaking of bonds within or between polymers as part of remodeling. In contrast, secondary cell walls are typically deposited only around fully expanded cells, adding strength, hydrophobicity, and a thick barrier for defense. Primary cell wall polymers during expansion are all polysaccharides which allow for different modes of remodeling; whereas, secondary cell wall polymers often include lignin where cross-links are considered irreversible. This review focuses on a particular subset of cell wall polymer modifications that occur on both polysaccharides and lignin, the abundant acylation with hydroxycinnamates that are a key feature of both primary and secondary cell walls of grasses and other commelinid monocots. Hydroxycinnamates are simple phenylpropanoid molecules, closely related to canonical lignin monomers, that share their ability to oxidatively couple and thereby cross-link polymers (Ralph et al., 1992; Ralph et al., 1995). The two most abundant cell wall phenolic esters in grasses, those derived from ferulic and *p*-coumaric acids (**Figure 1A**), differ greatly in this property. Feruloyl modifications (FA) have a much greater propensity than *p*-coumaroyl modifications (*p*CA) to undergo oxidative coupling (**Figure 1B, D**). This key difference has profound implications for the effects of these modifications on cell wall and biomass properties.

### Hydroxycinnamate modification of xylan

**Figure 2:**
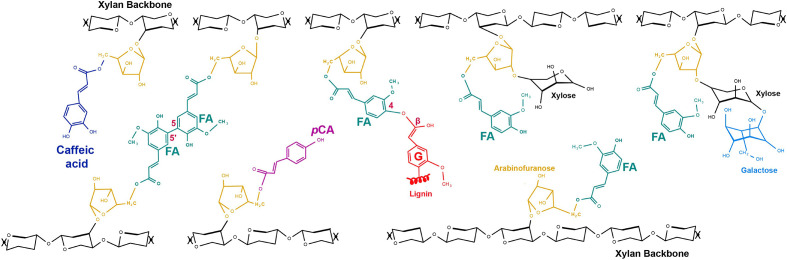
Hydroxycinnamoyl (HCA) decorations of grass glucuronoarabinoxylan (GAX). All HCA decorations occur on 5-O of Ara*f* which is α-(1,3)-linked to xylan backbone. FA decorations of GAX (turquoise) are abundant and include forms where Ara*f* is additionally substituted with β(1,2)-linked Xyl and this may itself be further substituted by β(1,4)-linked galactose. A FA 5,5’ dimer crosslinking GAX chains is shown as one example of a dimer that can crosslink GAX chains (see **Figure 1**). FA crosslinked to S lignin monomer is shown as one example of FA crosslinking GAX to lignin; FA can also link to G lignin monomers and to tricin (structures in **Figure 3**). *p*CA decorations (pink) of GAX can also be abundant in some tissues and smaller amounts of caffeoyl- decorations (dark blue) have recently been detected (Feijao et al., 2022). Glucuronic acid, O-methyl-glucuronic acid, acetyl substitutions, non-acylated Ara*f* that are commonly present on GAX are not shown here.

In grass primary and secondary cell walls, hydroxycinnamate modifications of polysaccharides occur as acylation of the 5-carbon of arabinofuranosyl (Ara*f*) decoration of the xylan backbone in glucuronoarabinoxylan (GAX; **Figure 2**) (Ishii, 1997; Ralph et al., 1998; Ralph et al., 2004; Buanafina, 2009; Bartley et al., 2013). Xylan is the most abundant polysaccharide other than cellulose in both primary and secondary cell walls of grasses, accounting for ~50% of grass hemicellulose (Scheller and Ulvskov, 2010), which represents, for example, 20-25% of dry switchgrass biomass (David and Ragauskas, 2010). Xylan is much more abundant in primary cell walls (PCWs) of grasses than in dicots (~30% compared to 5% of cell wall, respectively), displacing pectins as the most abundant non-cellulose polysaccharide. The 3-linked Ara*f* decoration of xylan is rare or absent in dicots and the FA and *p*CA acylation of this Ara*f* in GAX are believed to be completely specific to grass and other recently evolved monocots, known as commelinids (Harris and Trethewey, 2010). Grass GAX also possesses other substitutions on Ara*f*, such as β-(1->2)Xyl-(1->2)Gal (Saulnier et al., 1995), β-(1->2)-Gal and β-(1->2)-Xyl (Wende and Fry, 1997; Chiniquy et al., 2012), and substitutions shared with dicot xylan, such as acetylation and (4-O-methyl-) glucuronosyl at the O2- position (Scheller and Ulvskov, 2010). Other HCAs also occur at lower abundance ester-linked to GAX in grasses. Recent mass spectrometry analysis of products of mild acidolysis of rice cell walls has detected caffeic acid on Ara*f* of GAX (Feijao et al., 2022), and sinapate also occurs ester-linked to arabinoxylan in cereal grain (Bunzel et al., 2003). The presence of FA on GAX in particular confers a mode of cross-linking to grass primary cell walls absent in those of dicots since FA can undergo radical oxygen-mediated coupling to form ether bonds or C-C bonds, making diferulates and triferulates that result in xylan-xylan cross-linking (Takahama and Oniki, 1994; Bunzel et al., 2008) (**Figure 1C**). Across studies in various species and organs (Hatfield et al., 1999; Bartley et al., 2013; de Souza et al., 2018), the 8-5 and 8-O-4 dimers are often the most abundant diferulates (**Figure 1E**).

In PCWs, the functions of hydroxycinnamate modifications on GAX remain to be fully elucidated. The greater abundance of GAX and lower abundance of pectin and xyloglucan in grass PCWs compared to dicot PCW suggests grass GAX may have taken over some of the roles performed by pectin and xyloglucan, which is supported by solid-state NMR analysis of PCWs (Wang et al., 2014b). Potentially, the FA dimer and trimer cross-links on grass GAX partially substitute for the roles played by ionic cross-linking of pectin and for the oxidative cross-linking of extensin proteins in dicot PCWs. Consistent with this, the simplest form of extensins, those lacking a signaling domain, are not found in grasses; (Johnson et al., 2017). Also, FA is especially abundant per mass cell walls in very young tissue (Obel et al., 2002; Lin et al., 2016). Furthermore, abundance of cell wall FA and FA dimers was found to be negatively correlated with cell wall extensibility in wheat coleoptiles, suggesting an important role in control of PCW expansion (Wakabayashi et al., 1997). Additionally, hydroxycinnamates have antimicrobial properties (Akin, 2008) so their presence in grass primary cell walls also may serve to inhibit microbial attack and FA dimers (**Figure 1B, D**) may inhibit digestion (Grabber et al., 1998). Thus, the hydroxycinnamates on GAX in grass PCWs may confer evolutionary advantages by making young grass tissue with many expanding cells less readily digestible.

In lignified secondary cell walls (SCW) of both grasses and dicots, solid-state NMR suggests xylan in a twofold screw conformation (Xn^2f^) binds to cellulose microfibrils (Simmons et al., 2016); whereas, distorted twofold or threefold screw xylan (Xn^3f^) interacts closely with lignin (Kang et al., 2019; Duan et al., 2021). Thus, xylan bridges the two main components of SCW, although a study on sorghum SCW suggested Xn^2f^ was much less prevalent there (Gao et al., 2020). A clear difference in grass compared to dicot SCW is that the FA on GAX covalently bonds lignin *via* oxidative coupling, although the extent of this is difficult to determine (Ralph, 2010). GAX-FA is abundant on both Xn^2f^ and Xn^3f^ conformations in Brachypodium stems, and the authors proposed a model of grass SCW where FA on Xn^2f^ bound to cellulose crosslinks with other xylan FAs, and FA on Xn^3f^ covalently links to lignin (Duan et al., 2021). This model fits with several lines of evidence that show the abundance of GAX-FA and linkage of FA to lignin are correlated with recalcitrance to digestion of grass biomass (reviewed in (Buanafina, 2009; de Oliveira et al., 2015; Terrett and Dupree, 2019)). Thus, the FA-mediated linking of GAX to lignin inhibits access of hydrolytic enzymes to the cellulose to release glucose (the normal measure of digestibility).

In addition, a key role of FA in initial deposition of lignin is suggested by abundant Ara*f*-FA coupled to coniferyl alcohol, the G-lignin monomer released from grass SCW by mild acidolysis (Lapierre et al., 2019; Feijao et al., 2022). This supports a model that GAX-FA act as the nucleation sites from which the lignin polymers grow that was developed from biomimetic studies of lignification of maize suspension culture cell walls (Grabber et al., 2002). Interestingly, this mode of nucleation with many separate sites (i.e. abundant GAX-FA) may explain the lower molecular weight of grass lignin polymers compared with those of other plants, which could allow some flexibility in developing tissue (Hatfield et al., 2017).

The role of *p*CA on GAX is less apparent than that of FA because *p*CA oxidatively couples much less readily than FA and whereas FA-GAX is found in every tissue in grasses, *p*CA-GAX has low abundance in stems (Fanelli et al., 2021; Möller et al., 2022). One possibility is that, analogous to the putative role of *p*CA on lignin discussed below, *p*CA-GAX may participate in radical transfer, thus catalyzing the oxidative coupling of neighboring FA on GAX. This is compatible with the observation that *p*CA on GAX rapidly increases in response to jasmonic acid application to Brachypodium callus (Hyde et al., 2018) which could be part of a priming of defense, allowing rapid cross-linking to occur in response to additional signals.

### Hydroxycinnamate modification of lignin

**Figure 3:**
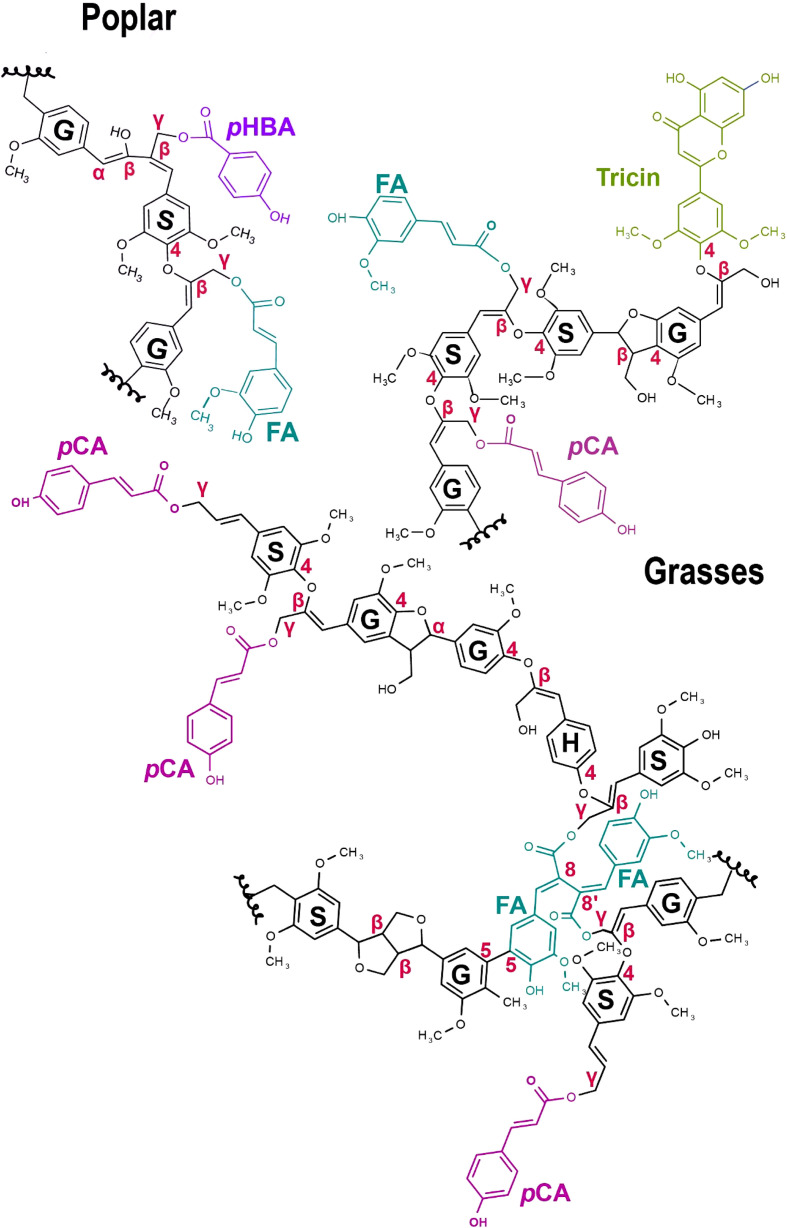
Hydroxycinnamoylated grass and poplar lignin polymer models. *p-*Coumaric acid (*p*CA - pink) and Ferulic acid (FA – turquoise) occur on lignin in commelinid grasses. Tricin (green) is a grass-specific flavonoid biosynthetic product that occurs as pendant groups on lignin. *p*-hydroxybenzoate (*p*HBA - purple) and FA (at low levels) occur in poplar and other dicots. γ-Feruloylated lignin naturally occurs in some non-commelinid grasses and dicots, generally at lower levels (Karlen et al., 2016). Here, we have only shown a FA dimer. Canonical lignin monomers include S, Syringyl lignin; G, Guaiacyl lignin; and H – *p*-hydroxyphenyl lignin. SGH monomers in the figure do not represent their actual ratios. The different C-C, ether (β-O-4), and γ-ester bonds occurring in the lignin polymer are highlighted in red.

Lignin biosynthesis occurs by generation of three main monolignols (*p*-coumaryl alcohol, coniferyl alcohol, and sinapyl alcohol) in cytosol and subsequent radical coupling of these in the apoplast (Boerjan et al., 2003). Monolignols acylated by phenolic acids (especially *p*CA; FA; and *p*-hydroxybenzoate, a simple phenolic with two fewer carbons than hydroxycinnamates) and acetate, are now established as additional monomers of lignification in various species (**Figure 3**). Attachment of *p*CA to lignin has been found in a diversity of grass species (Soreng et al., 2015) including maize, bromegrass, bamboo, sugarcane, elephant grass, rice (Withers et al., 2012; Karlen et al., 2016; Takeda et al., 2017), switchgrass (Shen et al., 2009), and Brachypodium (Petrik et al., 2014). Recently, *p*-coumaryl lignin was also found in other commelinid monocots (*Zingiberales, Commelinales, and Arecales*) (Karlen et al., 2018) and in the dicot mulberry (Moracacea) (Hellinger et al., 2022). Another phenolic acid, *p*-hydoxybenzoate, also occurs ester-linked to lignin in the poplar, willows, and oil palms (de Vries et al., 2021; Zhao et al., 2021). Like *p*CA, *p*-hydoxybenzoate does not readily oxidatively couple, so terminates lignin chains (**Figure 3**). The acylation of monolignols by the other major hydroxycinnamate, FA, is a topic of great biotechnological interest because the FA becomes incorporated into lignin polymer *via* its propensity to oxidatively couple, thereby introducing alkaline-labile ester bonds (**Figure 3**) making the lignin much easier to break apart (Wilkerson et al., 2014). We discuss this further in biotechnological applications below. It is now clear that FA-lignin occurs at low abundance naturally in all commelinids examined as well as sporadically within eudicots (Karlen et al., 2016).

The role of lignin acylation by hydroxycinnamates is uncertain. One possibility is that *p*CA (and hydroxybenzoate) moieties on lignin act as “radical catalysts.” Model studies of *p*CA show that it is readily oxidized. However, the fact that it has not been observed to oxidatively couple *in muro* has led to a model that oxidized *p*-coumaryl esters rapidly pass radicals to sinapyl alcohols, thereby facilitating lignin polymerization (Takahama and Oniki, 1994; Ralph, 2010). For the lower abundance acylation by FA, the biological functions are an open question.

## BAHD acyl CoA transferases

We have gradually gained knowledge of enzymes responsible for the incorporation of *p*CA and FA into grass cell walls on both GAX polysaccharide and lignin. These proteins are all “BAHD” acyl-CoA acyltransferases, a large enzyme family in plants that acylate metabolites with CoA thioester donors named for the first four activities described for this family (BEAT, AHCT, HCBT, and DAT) (D'Auria, 2006). The BAHD family is divided into five clades; Clade V includes quinate hydroxycinnamoyl transferase (HCT) an enzyme in phenylpropanoid pathway for monolignol synthesis. BAHD enzymes are known for their versatility (i.e., low specificity) and often show activity with multiple acyl-CoA donors and acceptors such that their activity *in vivo* might be dictated by relative availability of substrates (D'Auria, 2006). They are also known for examples of convergent evolution as BAHD enzymes from different Clades can have the same activity (Luo et al., 2007).

### Candidate BAHD enzymes for feruloylation and p-coumarylation of GAX

**Figure 4:**
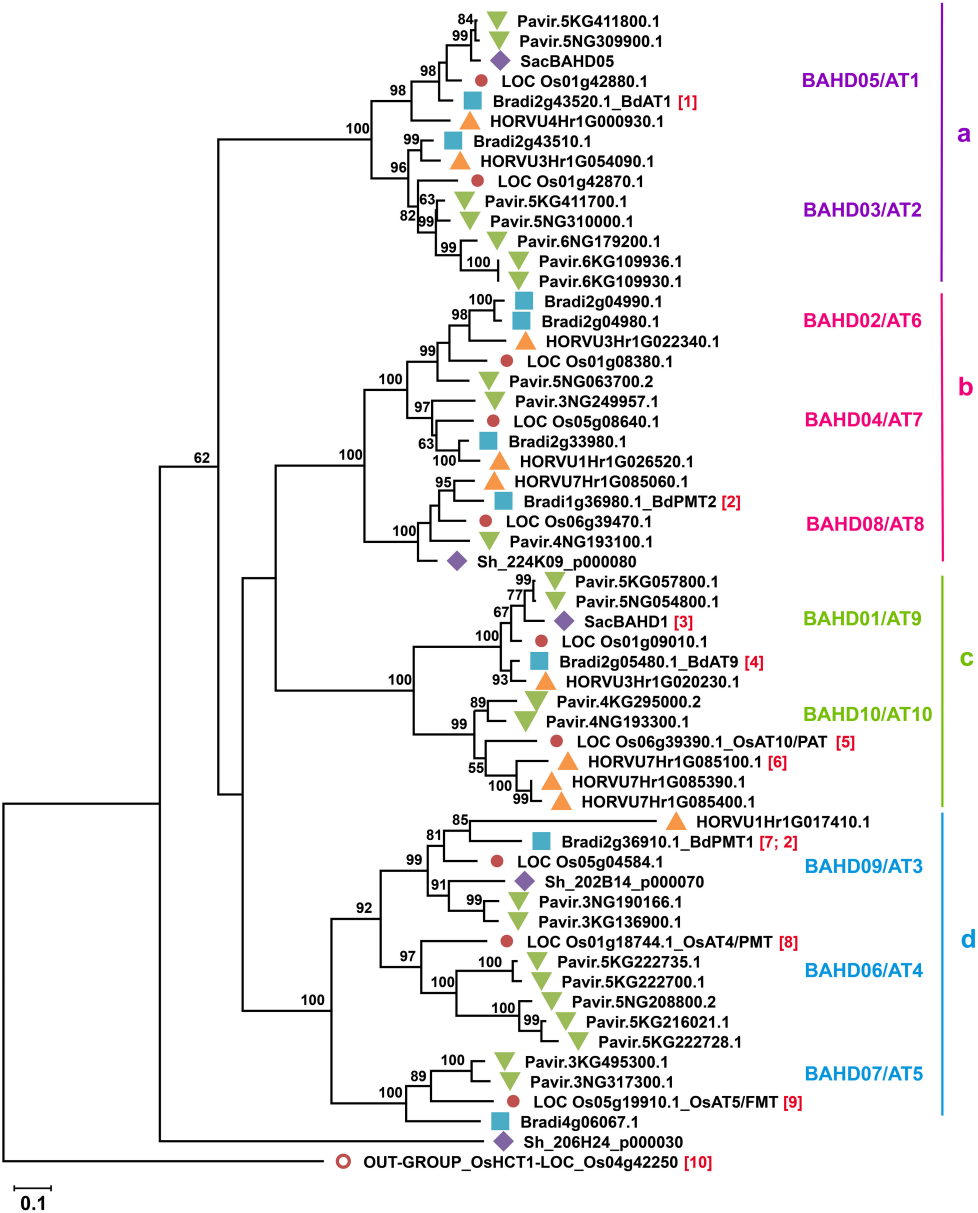
Phylogenetic reconstruction of Mitchell subclade of grass BAHD acyltransferases. Phylogenetic tree was generated by maximum likelihood method (Jones et al., 1992) using Mega X software (Kumar et al., 2018) after multiple sequence alignment by MUSCLE (3.8). All sequences of rice genes (Os) originally identified in Mitchell subclade (Bartley et al., 2013; Molinari et al., 2013) and their orthologs in *Brachypodium distachyon* v3.1 (Bradi), *Saccharum* (Sh; sequences from (de Souza et al., 2019), *Hordeum vulgare* (HORVU) and *Panicum virgatum* v5 (Pavir) are included. The rice HCT gene OsHCT1 Kim et al. (2012) [10] was used as an outgroup. The division of proteins into sub-groups (a-d) as in Karlen et al. (2016) is indicated on the right. Proteins that are functionally characterized are as follows: [1] Buanafina et al. (2016), [2] (Sibout et al., 2016), [3] de Souza et al. (2019) [4] de Souza et al. (2018) [5] Bartley et al. (2013) [6] Houston et al. (2020), [7] Petrik et al. (2014), [8] Withers et al. (2012), [9] Karlen et al. (2016). Where assigned, enzyme activities are PMT *p*-Coumaryl CoA Monolignol Transferase, FMT Feruloyl CoA Monolignol Transferase, PAT *p*-Coumaryl CoA Arabinoxylan Transferase. Numbers on tree nodes are percentage bootstrap support; values <50 not shown. Scale bar indicates branch lengths measured in the number of substitutions per site.

**Figure 5:**
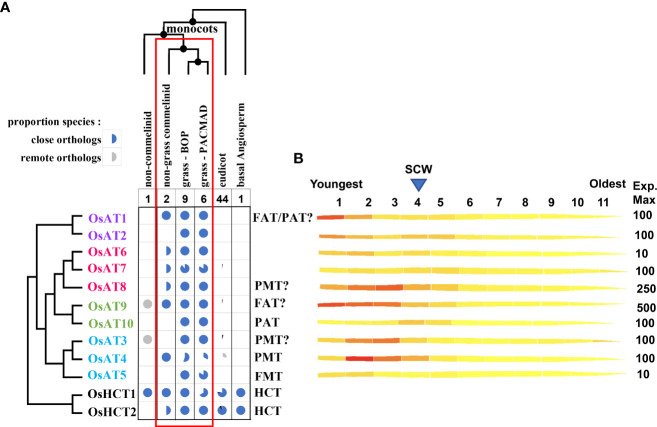
Taxonomy and expression profiles of Mitchell subclade acyltransferases. **(A)** Taxonomic distribution of ATs and their known and putative activities. Pie charts indicate the proportion of species with orthologs to the Mitchell subclade OsAT genes and to two BAHD Clade V HCT genes encoding shikimate O-hydroxycinnamoyl transferases, a key enzyme in the phenylpropanoid pathway expected to be present in all plants. All species have close orthologs to HCT1 and/or HCT2 whereas only commelinid monocots have close orthologs to *AT* genes, which matches the taxonomic distribution of FA-GAX indicated by the red rectangle. The species are all angiosperms with fully sequenced genomes present in Ensembl Plants release 54; close and remote orthologs defined as reciprocal blastp top hits with bitscore > 400 and 300 respectively. Text colors indicated the subgroups in **Figure 4**. FAT indicates likely feruloyl arabinose transferase; PAT indicates likely *p*-coumaryl arabinose acyltransferase; FMT indicates ferulate monolignol transferase; PMT indicates *p*-coumarate monolignol transferase. Question marks indicate that existing evidence is contradictory or relatively weak. **(B)** *AT* expression across the rice leaf gradient generated from the eFP (electronic fluorescent pictograph) browser of the Bio-Analytic Resource for Plant Biology (BAR), the University of Toronto (Sullivan et al., 2019) using Wang et al. (2014a) leaf expression data. The rice leaf gradient includes the youngest tissue in the 1^st^ segment and the oldest in the 11^th^ segment with secondary cell wall (SCW) related expression peaking at segment 4.

Looking for candidate genes for addition of FA to GAX, Mitchell et al. (2007) searched for genes that are highly expressed in grasses while the most similar genes in dicots are much less expressed and differ substantially in protein sequence, since feruloylation is abundant in every grass tissue and absent in dicots. They found a small subclade of BAHD genes that met these criteria in Clade V [Clade Va of Tuominen et al. (2011)] and as acyl transferases these were postulated as involved in feruloylation. Furthermore, some of these BAHD grass genes are co-expressed with other genes responsible for GAX synthesis (Mitchell et al., 2007; Molinari et al., 2013). To facilitate communication about these grass BAHD acyltransferases, Bartley et al. (2013) called the group of 20 rice genes the “Mitchell Clade” and identified subclade i and subclade ii containing, *Oryza sativa* (Os) acyltransferases (AT), OsAT1-OsAT10, and OsAT11-OsAT20, respectively. **Figure 4** shows subclade i for selected model and economically relevant grass species. Alternative names were proposed of the form BAHD01-BAHD20 (Molinari et al., 2013) and are used in some publications; here we show the equivalent names in **Figure 4** but will use the AT nomenclature in the text. As discussed in greater detail below, grasses generally possess 8-10 subclade i ATs per haploid genome (**Figures 4**, **5**). The function of Mitchell subclade ii genes, which have undergone grass species-/tribe-specific expansions/deletions (Karlen et al., 2016) remains unknown. Due to the absence of studies about them and their generally low expression (Bartley et al., 2013) we have excluded the subclade ii genes from this review and use “Mitchell subclade” to refer exclusively to subclade i.

There is now good evidence that several Mitchell subclade ATs are indeed involved in acylation of GAX, as predicted; while others acylate monolignols. Genetic manipulation of the Mitchell subclade was first achieved by Piston et al. (2010) by simultaneous downregulation of OsAT7, OsAT8, OsAT9, and OsAT10 in rice which resulted in decreased amounts of ester-linked FA in a cell-wall enriched fraction from leaves. The authors observed 2- to 3-fold reductions in gene expression of these ATs and an average of 20% reduction in cell wall FA content in the leaves, but due to use of constructs targeting multiple ATs could not determine which of the silenced genes were responsible and did not demonstrate that the FA was attached to GAX. Bartley et al. (2013) provided the first genetic evidence on single ATs involved in addition of hydroxycinnamates to GAX. Using mild acidolysis to break glycosidic bonds, they were able to show effects on a five-carbon sugar-esterified hydroxycinnamates, likely Ara-*p*CA and Ara-FA, released from rice cell walls (rather than saponification to release ester-linked FA and *p*CA from cell wall polymers, in general). They designated OsAT10 as a putative *p*-coumaroyl CoA arabinofuranose transferase (PAT) since overexpression of *OsAT10, via* an activation tagged line in rice (*OsAT10-D1*), induced a 5-fold increase in *p*CA levels in young green tissues. The observed increased saccharification yields, in the transgenic line were possibly due to concomitant 50% decrease in FA linked to GAX (Bartley et al., 2013). Since then, other studies also achieved several-fold increases in *p*CA-GAX by heterologous expression of *OsAT10* in switchgrass (Li et al., 2018) and sorghum (Tian et al., 2021) and of sugarcane *AT10* (*ScAT10*) in maize (Fanelli et al., 2021). As in Bartley et al. (2013) this was sometimes (Li et al., 2018; Fanelli et al., 2021), but not universally, accompanied by a decrease in FA-AX. Assumed to be an indirect effect, the mechanism of the alternating abundance of *p*CA-AX and FA remains an open question. Since then, a complete knock out mutants of *OsAT10* in rice have been generated using CRISPR/Cas9 rice plants, leading to an almost complete lack of *p*CA-GAX, which was found to be most abundant in rice husks, compared to mature leaf and stems, of wild-type plants (Möller et al., 2022). The gene edited AT10 lines also exhibited an increase in FA, but no differences in cell wall composition or digestibility. Giving further support for the AT10 PAT function across species, a natural allele of the *AT10* ortholog in barley (*HORVU7Hr1G085100*; **Figure 4**), predicted to encode a defective enzyme, has less ester-linked *p*CA and more ester-linked FA in grain cell walls (Houston et al., 2020).

Genetic analysis has provided various strengths of support for several ATs acting as putative feruloyl arabinofuranose transferases (FATs). RNAi silencing of Brachypodium *BdAT1* showed an approximately 25% reduction in FA amounts and BdAT1 overexpression resulted in an approximately 15% increase in FA in leaves and stems (Buanafina et al., 2016). On the other hand, Mota et al. (2021) showed different results, with RNAi suppression of *SvAT1*, the *Setaria viridis* BdAT1 ortholog, decreasing *p*CA not FA on GAX. They therefore suggest that BdAT1 and SvAT1 have differing specificities for *p*CA-CoA and FA-CoA donors and conduct some protein structural modelling to support this. The clearest evidence of FAT activity thus far, was obtained through RNAi-based silencing of *SvAT9* (*SvBAHD01*) in Setaria resulting in a 60% decrease in FA-GAX with a significant increase *p*CA-GAX; whereas, downregulation of *OsAT9* ortholog in Brachypodium showed only small effects on FA (de Souza et al., 2018). *AT7* ortholog downregulation in Brachypodium did not yield any significant changes in FA (Buanafina et al., 2016), though preliminary evidence suggested that a rice T-DNA insertion line for this gene has less leaf sheath FA (Bartley et al., 2013). In general, whilst genetic manipulation of ATs in grasses has achieved many fold increases and abolition in some tissues of *p*CA-GAX, this has not been reported for FA-GAX where the range of effects is narrower. This may point to a critical functionality of FA-GAX in grass cell walls where abolition would be lethal and large increases are difficult to achieve due to tight regulation.

### BAHD enzymes acylate lignin monomers

Independent of the bioinformatics identification of the Mitchell subclade as candidates for GAX feruloylation, other groups established that some members of this subclade add *p*CA to lignin by acylating monolignols. Withers et al. (2012) showed that OsAT4 functions *in vitro* as a *p*CA monolignol acyltransferase (PMT) that transfers *p*CA from *p*CA-CoA onto H and S monolignols. The maize ortholog of *OsAT3* also shows PMT activity, and RNAi suppression of the maize led to large decreases in *p*CA ester-linked to lignin (Marita et al., 2014). The strongest line showed a reduction in S lignin. Similarly, a complete knock-out mutant of *BdPMT1*, the Brachypodium ortholog of *OsAT3*, had <0.5% *p*CA on mature lignin; whereas *p*CA on GAX was unaffected. Conversely, overexpression of *BdPMT1* boosted *pCA-*lignin above wild-type levels (Petrik et al., 2014). Heterologous expression of *BdPMT1* and *BdPMT2* (ortholog of *OsAT8*) in *Arabidopsis*, under the control of the *Arabidopsis* cinnamate-4-hydroxylase promoter, introduced *p*CA onto lignin, showing a gain of function since there is no *p*CA on lignin in wild-type Arabidopsis (Sibout et al., 2016). Though not focused on lignin modification, an early study found an enzyme from the commelinid species, *Musa sapientum* (i.e., banana alcohol acyltransferase, BanAAT), to have the highest activity on an aromatic acceptor substrate (Beekwilder et al., 2004). In retrospect, based on phylogenic analysis (Bartley et al., 2013), this enzyme is likely a banana PMT.

BAHD enzymes that acylate monolignols with FA have also been discovered. A feruloyl-monolignol transferase (FMT) from Chinese angelica [*Angelica sinensis* (*As*), a dicotyledonous medicinal plant, was heterologously expressed in hybrid poplar generating monolignol-FAs that were incorporated into lignin polymers (Wilkerson et al., 2014). The use of this activity to facilitate cell wall deconstruction are discussed further below (see Biotechnological Applications). The AsFMT is in Clade III of the BAHD superfamily, which is distant from the Mitchell subclade within Clade V. Surprisingly, overexpression of *OsAT5* in rice increased feruloylated monolignols, suggesting that *OsAT5* also encodes an FMT (Karlen et al., 2016). Thus, AsFMT and OsFMT are the result of convergent evolution, one of several examples in the BAHD family (Luo et al., 2007). A recent discovery on substrate specificity was made by Smith et al. (2022) looking at FMT and PMT enzymes from sorghum (*Sorghum bicolor*) and switchgrass (*Panicum virgatum*) as synthesized with wheat germ extract followed by *in vitro* characterization. The FMT enzymes, including OsAT5, produced both monolignol FA and monolignol *p*CA conjugates; whereas, the PMT enzymes produced exclusively monolignol *p*CA conjugates. A tolerance of differing acyl-CoA donors is another known feature of many BAHD enzymes (D'Auria, 2006).

## Taxonomic distribution and patterns of expression of *AT* genes

The taxonomic distribution and expression of *AT* genes provide functional clues and be used to identify other candidate genes involved in the same processes for basic and applied purposes. Phylogenetic analyses from selected grass species here (**Figures 4**, **5**) and elsewhere (Bartley et al. (2013); Karlen et al. (2016); de Souza et al. (2018); Fanelli et al. (2021)) shows that the Mitchell subclade ATs are highly conserved in grasses. Here, we identified orthologs from a novel set of species to better assess how their distribution compares that with that of FA and *p*CA ester-linked GAX and lignin discussed above. The distribution of orthologs of the Mitchell subclade ATs are shown in **Figure 5** and their corresponding demonstrated and putative activities noted. As outlined above, FA-GAX is likely a fundamental feature of grass cell walls, conferring a mode of cross-linking absent in cell walls of plants outside of the commelinid monocots which plausibly represents a trait that contributed to the evolutionary success of the grasses. Therefore, we might expect the enzymes responsible to be highly conserved in all grasses. Consistent with this, in fully sequenced genomes of 15 grasses, all have clear orthologs to *OsAT1*, *OsAT2*, *OsAT3*, *OsAT6*, *OsAT8*, *OsAT9*, and *OsAT10* (**Figure 5**). Thus, genes demonstrated to be responsible for acylation of GAX with FA and *p*CA and of lignin with *p*CA in some grasses are conserved, suggesting conservation of these functions across the Poaceae. Indeed, biochemical analysis of sorghum and switchgrass orthologs of rice and Brachypodium monolignol ATs, supports the notion that sequence conservation indicates functional conservation, albeit with variation in enzymatic parameters (Smith et al., 2022). However, the absence of conservation of AT5 suggests that either the FMT activity may be dispensable, or another AT may have this activity, either primarily or due to low substrate specificity.

Looking more broadly across monocots, there are also clear orthologs of Mitchell subclade ATs in non-grass commelinids (*Musa acuminata* and *Ananas comosus*) of one member of each enzyme group (a-d), i.e., OsAT1, *OsAT3*/4, OsAT6, OsAT9. The occurrence of close orthologs thus matches the distribution of GAX feruloylation, believed to be confined to commelinid monocots (Harris and Trethewey, 2010). As described above, *p*CA-lignin occurs in all commelinid monocots examined by Karlen et al. (2018) but has also recently been reported in the eudicot mulberry (Hellinger et al., 2022) but mulberry is not within set of eudicot genomes used in **Figure 5**. None of analyzed species outside the commelinid monocots encode close orthologs to the AT proteins, but there are some remote orthologs to *OsAT3* and *OsAT9* detected in the non-commelinid monocot, *Dioscorea rotundata*, and of *OsAT3*, OsAT4, and OsAT7 and OsAT9 within eudicots. This suggests the origin of Mitchell clade was a gene present in the common ancestor to monocots and eudicots that underwent sequence divergence and gene duplication first in commelinid moncocots and then further in grasses, whereas the genes were lost in most eudicots.

The distribution of cell wall hydroxycinnamates is, however, known to be broader than that of the Mitchell subclade genes. Karlen et al. (2016) showed that whilst feruloylated lignin occurs in all grasses tested, it is also detected in dicots like poplar, balsa, aspen, red maple, Babylon willow, eucalyptus, hibiscus, and *Angelica sinensis*. As those authors discussed, this is likely due to convergent evolution of other unrelated BAHDs such as AsFMT. FA also occurs as a cross-linking moiety on pectin in cell walls of dicots in order *Caryophyllales*, e.g. spinach (Fry, 1986), and FA and *p*CA have both also been reported in primary cell walls of gymnosperms ester-linked to an unknown component (Carnachan and Harris, 2000). Another unrelated BAHD has recently been shown to be responsible for the acylation of lignin with the phenolic acid *p*-hydroxybenzoate in poplar (de Vries et al., 2021; Zhao et al., 2021). While convergent evolution of addition of hydroxycinnamates and similar phenolics to cell wall polymers therefore appears widespread in seed plants, to-date, hydroxycinnamates acylation of GAX appears to be confined to commelinid monocots.

The Mitchell subclade ATs fall into four groups (a-d, **Figure 4**) due to multiple small differences in sequence. Group ‘d’ contains the studied hydroxycinnamate monolignol transferases (AT3, AT4, AT5) and ‘c’ contains the apparent GAX-transferases (AT9 and AT10). Thus, enzymes within groups ‘d’ and ‘c’ likely act on common acceptor substrates, but varied CoA donors. The other two groups contain less well- or un-characterized genes and furthermore, the bootstrap support from the phylogenetic analysis does not position group ‘a’ confidently relative to the others (**Figure 4**).

Gene expression data can hint at roles of ATs, particularly relative to their function in synthesis of PCW and SCW in grasses. **Figure 5B** illustrates Mitchell subclade AT gene expression in rice leaf [from (Wang et al., 2014a)] along a developmental gradient, from the intercalary meristem at the leaf base (segment 1, on the left), an elongation zone (segments 2 and 3), to the transition to SCW formation [approximately segment 4, based on peak expression of SCW-inducing transcription factors (Li et al., 2010)]. *OsAT3* and *OsAT4*, encoding the PMTs, possess similar profiles that peak just in segments 2 and 3, respectively. Consistent with its assignment as another PMT (Sibout et al., 2016), this is also the pattern of expression for *OsAT8* (group d), which shows the second highest transcript abundance among the Mitchell subclade. On the other hand, *OsAT9*, which shows the highest transcript abundance in the clade, is highest in segment 1, where mostly PCW synthesis occurs, and continues to be abundant until past the SCW peak. *OsAT1* (group a), which has also been tentatively assigned as a FAT (Buanafina et al., 2016), shows a similar pattern. Potentially with implications for a particular function of the *p*CA-GAX modification later in development, *OsAT10* displays the latest expression peak, at segment 4. By contrast, consistent with a potential role in maintaining lignin flexibility/lability early in development (i.e. in still elongating vascular cells), putative FMT, *OsAT5*, which is among the lowest expressed of the Mitchell subclade in the leaf, peaks early in development. The uncharacterized ATs (*OsAT2*, *OsAT6*, and *OsAT7*) all exhibit a similar double peak of expression, with an initial peak in segments 1 or 2 and a second peak in segments 4 or 5, suggesting these genes might function in both PCW and SCW synthesis (Lin et al., 2016).

## Models for the mechanism of incorporation of HCAs into lignin and xylan

### FA on GAX may derive from a different pool of phenylpropanoids than *p*CA on lignin

**Figure 6:**
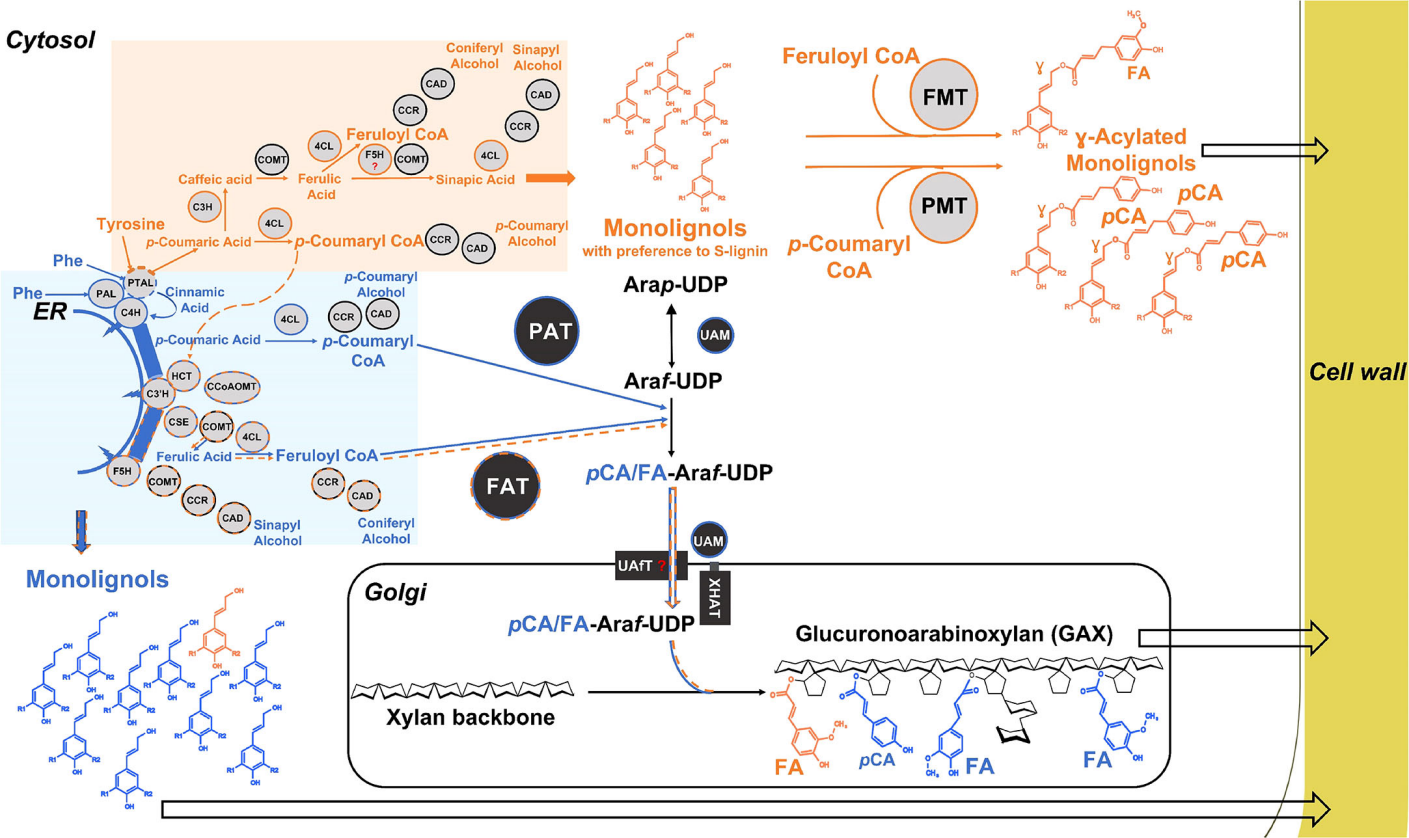
Model of hydroxycinnamoylation of lignin and GAX in grasses. The model shows putative pathways for the synthesis of HCAs and monolignols and their routes into the grass cell wall. The phenylalanine precursor-based primary phenylpropanoid pathway with early steps catalyzed by ER-localized enzymes is highlighted in blue and the orange color represents the tyrosine precursor-based pathway; some enzymes (4CL, CAD, CCR, COMT) are shared between these pathways but are shown separately for clarity. Dashed lines are our speculations. Question marks are added for the cytosolic F5H and UAfT as they have yet to be identified. γ-acylated monolignols: S lignin – R1 = R2 = OMe, G lignin – R1 = OMe, R2 = H. Acyltransferases – PMT, *p-*Coumaryl CoA Monolignol Transferase, FMT, Feruloyl CoA Monolignol Transferase; PAT, *p-*Coumaryl CoA Arabinoxylan Transferase; FAT, Feruloyl CoA Ara*f* transferase; Lignin biosynthetic enzymes – PAL – monofunctional Phenylalanine Ammonia-Lyase, PTAL, bifunctional Phenylalanine/Tyrosine Ammonia-Lyase; C4H, Cinnamate 4-Hydroxylase; C3’H - 4, Coumaroyl Shikimate/Quinate 3-Hydroxylase, HCT - Hydroxycinnamoyl CoA Shikimate/Quinate Hydroxycinnamoyl Transferase, 4CL - 4-Coumarate : CoA Ligase, F5H1, Ferulate 5-Hydroxylase; C3H - bifunctional 4-Coumarate 3-Hydroxylase/Cytosolic Ascorbate Peroxidase, COMT - Caffeic Acid/5-Hydroxyferulic Acid 3/5-O-Methyltransferase, CSE, caffeoyl shikimate esterase; CCoAOMT, caffeoyl CoA 3-O-methyltransferase; CCR, cinnamoyl CoA reductase; CAD - cinnamyl alcohol dehydrogenase; GAX related enzymes – UAM – UDP-arabinose mutase, UAfT, UDP-Ara*f* transporter; XHAT, xylan (hydroxycinnamoyl)-Ara*f* transferase; ER, Endoplasmic reticulum.

The *p*CA-CoA and FA-CoA molecules that act as donors for the ATs are metabolites within the phenylpropanoid pathway that synthesizes monolignols. In grasses, recent evidence points to the presence of two largely separate *p*CA-CoA pools derived from the two phenylpropanoid pathway precursors phenylalanine and tyrosine (Barros et al., 2016; Wang et al., 2018; Simpson et al., 2021; Barros et al., 2022). Current thinking is that endoplasmic reticulum (ER)-associated cytochrome P450 enzymes, C4H, C3′H, and F5H, form a metabolon with the soluble enzymes, PAL, 4CL, HCT, facilitating metabolic channeling (Winkel, 2004; Bassard et al., 2012; Gou et al., 2018; Zhang et al., 2022). (See the legend of **Figure 6** for the enzyme acronyms used here.) PTAL, a bifunctional enzyme, might also be involved in metabolon formation with ER-associated enzymes. Initial evidence for multiple *p*CA pools is the observation that PTAL’s tyrosine ammonia-lyase activity provides half the total lignin in Brachypodium stems, and wall-bound *p*CA with minimal contribution to wall-bound FA (Barros et al., 2016). Further, downregulation of C3′H and F5H in rice decrease unacylated G/S-lignin but do not alter amounts of *p*-coumaroylated G- or S-lignin; C3′H downregulation also significantly decreases wall-bound FA (Takeda et al., 2017; Takeda et al., 2018; Takeda et al., 2019). Thus, one explanation for the observation that unacylated monolignols and FA on GAX and *p*CA-monoligols appear to require different enzymes, is that there are separate *p*CA pools, though other explanations, such as metabolic compensatation (Vanholme et al., 2012), is possible.

**Figure 6** summarize a model, which remains to be tested, of separate tyrosine and phenyalanine-derived hydroxycinnmate pools and partially distinct cell wall products. When tyrosine enters as a substrate to the PTAL, the product, *p*CA, is not utilized by C4H and hence escapes into the cytosol avoiding the initial metabolon channel. The pool of “escaped” *pCA* is utilized by the cytosolic enzymes C3H, COMT, 4CL, CCR, and CAD to produce a part of *p*CA-CoA, FA-CoA, and γ-hydroxycinnamoyl acylated monolignols. [C3H is a recently discovered cytosolic enzyme that directly catalyzes the 3-hydroxylation of 4-coumarate to caffeate, bypassing the previously known shikimate shunt involving C3’H and HCT (Barros et al., 2019)]. A part of the *p*CA-CoA and FA-CoA produced from the “escaped *p*CA” enters back to the monolignol pathway, which is supported both by the results with the PTAL mutant and the observation that heavy atom labeled tyrosine feeding studies in sorghum do result in labeled *p*-coumaryl shikimate (Simpson et al., 2021). Thus, these recaptured hydroxycinnamates can contribute to producing minor amounts of FA-CoA, utilized by FATs to substitute FA on GAX. In contrast, the phenylalanine precursor-based PAL/PTAL-ER-associated enzymes contribute to a major part of cell-wall-associated monolignols, FA-CoA, and *p*-CA-CoA which are utilized by FATs and PATs to decorate GAX. Recently, loss-of-function of two rice 4CL homologs, Os4CL3 and Os4CL4, differentially altered non-acylated and acylated monolignol content (Afifi et al., 2022, indicating divergent roles of 4CL protein isoforms and providing further support for the model. A final step of lignin acylation, it was recently hypothesized that monolignol-FA and monolignol-*p*CA are synthesized in the cytosol and exported into the cell wall by the same simple diffusion mechanism as monolignols (Vermaas et al., 2019).

### Mechanisms of hydroxycinnamoyl incorporation onto arabinoxylans

**Figure 6** also illustrates a plausible model for how Mitchell subclade ATs can be responsible for FA and *p*CA incorporation into arabinoxylans (AX). Biosynthesis of AX is carried out by glycosytransferases confined to the Golgi lumen where IRX9 and IRX10 proteins participate in a xylan synthase complex (Zeng et al., 2016) and grass XAT proteins mediate Ara*f* decoration (Anders et al., 2012). However, the BAHD ATs are known to be cytosolic, as expected from their sequences which lack transmembrane domains and secretory pathway sequences. In addition, their hydroxycinnamoyl-CoA substrates are cytosolic and not known to occur in the Golgi lumen. Therefore, it seems that F/PATs must acylate a cytosolic precursor to AX synthesis just as P/FMTs acylate cytosolic lignin precursors. This conclusion can also explain the apparently surprising early result that feruloylation activity was found in the cytosolic fraction, not the membrane fraction, of rice cell cultures (Yoshida-Shimokawa et al. (2001); the Ara*f*-Xyl*p*-Xyl*p* acceptor used there is presumably not the natural one but is sufficiently close to be recognized by an endogenous FAT).

The obvious candidate for the natural cytosolic AX precursor is UDP-β-L-arabinofuranose (UDP-Ara*f*) since the UDP-arabinose mutase (UAM) responsible for its generation is localized outside the Golgi lumen, either in the cytsosol or to the Golgi perihpheral region (Konishi et al., 2011; Rautengarten et al., 2011). This is believed to be the last cytosolic step, and UDP-Ara*f* would then be transported by a nucleotide sugar transporter (UAfT) into the Golgi lumen. Therefore, the simplest model is that cytosolic BAHD ATs catalyze the acylation of UDP-Ara*f* to give UDP-Ara*f*-FA/*p*CA as intermediates (**Figure 6**). However, these putative products have not been identified despite targeted searches in grass tissues. One possibility is that these metabolites are only stable when bound to proteins, being generated by the action of ATs on UDP-Ara*f* whilst this is still bound to UAM before it is transferred to the UAfT transporter (Hatfield et al., 2017). A protein complex involving both UAM and AT localized to Golgi periphery has been postulated (Hatfield et al., 2017) but proteomics from Brachypodium callus suggest that whereas UAM occurs both in peripheral and cytosolic fractions, ATs occur only in cytosol (JF and RACM, unpublished). One possibility is that UAM with bound UDP-Ara*f* shuttles from the Golgi periphery to the cytosol where acylation of UDP-Ara*f* occurs before returning to the periphery to engage with UAfT. This transporter could be similar to known UDP-Ara*f* transporters (Rautengarten et al., 2017) with variation that permits the FA/*p*CA modification, which are small in comparison to UDP. Sharing most of the machinery for generating FA and *p*CA acylated UDP-Ara*f* could also explain the apparent trade off in abundance of FA- and pCA-GAX in many experiments on different grass species when PAT or FAT expression is modified.

This model also necessitates a Golgi-localized GT enzyme to attach FA/*p*CA-Ara*f* to the growing xylan molecule i.e. a xylan (hydroxcinnamoyl)-Ara*f* transferase (XHAT; **Figure 6**). Addition of non-acylated Ara*f* to xylan is mediated by grass-specific enzymes in GT family 61 (Anders et al., 2012) and it was reported that a closely related GT61 enzyme was responsible for addition of a xylosyl residue to GAX, so this enzyme was named XAX1 (Chiniquy et al., 2012). However more recent LC-MS analysis of sugar products released by mild acid treatment from the rice *xax1* mutant suggests that XAX1 functions in the transfer of hydroxycinnamoyl-Ara*f* to xylan, as all FA-Ara*f* and *p*CA-Ara*f* decorations of GAX were decreased in the mutant compared with the wild type (Feijao et al., 2022). This study therefore provides strong evidence that XAX1 is an XHAT responsible for the incorporation of FA/*p*CA-Ara*f* onto xylan in the Golgi lumen.

Overall, the models in **Figure 6** highlight the similarity of action of PMT/FMT and FAT/PAT in acylating hydroxyl groups on, respectively, monolignols and the Ara*f* sugar in the cytosol, consistent with their similar primary sequences. Structural studies of these enzymes are required to understand the factors determining their specificities for acceptor and donor substrates.

## Catalytic mechanisms of Mitchell subclade acyltransferases

The major conserved domain shared by BAHD family enzymes contains a HXXXDG motif, located near the center portion of each enzyme, with the second highly conserved region being the DFGWG motif, located near the C-terminus (D'Auria, 2006). The first crystal structure of a BAHD enzyme, vinorine synthase, was obtained by Ma et al. (2005), making a large contribution to understanding the function of conserved domains that are shared among BAHD family members. In general, the proposed catalytic mechanism involves the histidine residue in the HXXXDG motif, which deprotonates the oxygen or nitrogen atom on the corresponding acceptor substrate, allowing a nucleophilic attack on the carbonyl carbon of the CoA thioester donor, which in turn forms a tetrahedral intermediate between the CoA thioester and the acceptor substrate. This intermediate is then reprotonated, giving rise to free CoA and the acylated ester or amide. This general catalytic mechanism has however not yet been confirmed for Mitchell subclade ATs, but generalized forms of bot motifs do occur as HXXXDG and D[FY]GXG motifs in them. Although no experimental structures have been reported for the Mitchell clade ATs, the convergently evolved AsFMT structure has been solved (Liu et al., 2022). The authors showed several unique structural features of AsFMT compared to other BAHD homologs, and molecular docking studies suggest that T375 in AsFMT may function as an oxyanion hole to stabilize the reaction intermediate. These studies also proposed a role of H278 in the binding of the nucleophilic hydroxyl group of monolignols.

## Biotechnological applications

Mature plant biomass, composed principally of SCWs and therefore termed lignocellulosic biomass, is a promising feedstock for production of next-generation fuels and chemicals that can replace fossil carbon sources thereby reducing greenhouse gas emissions (Farrell et al., 2006; Fargione et al., 2008; Schmer et al., 2008; Chundawat et al., 2011). To be economically and environmentally viable, this biomass can be non-food residues of crops (sugarcane bagasse, corn stover, paper mill waste and cereal straw) or from dedicated energy crops grown with minimal inputs. However, the cost and inefficiency of depolymerizing polysaccharides to fermentable sugars, also known as cell wall recalcitrance, are important impediments to large-scale lignocellulosic biofuel production (Lynd et al., 2008).

Manipulation of acylation of SCW polymers with ferulate, in particular, is a promising approach to improve the digestibility of biomass because it combines ester links with capacity for oxidative coupling, with opposite direction of effects depending on the polymer context. Feruloylation of GAX in grasses cross-links xylan strands to each other and to lignin, increasing recalcitrance. Conversely acylation of monolignols with ferulate results in the introduction of alkali-labile ester bonds into the body of the lignin polymer improving ease of saccharification, a technology referred to as “Zip-lignin”, by Ralph and colleagues (Wilkerson et al., 2014). Therefore, decreasing feruloylation of GAX in grass biomass and introducing or boosting feruloylation of monolignols in important biomass crops such as poplar are both promising biotechnological approaches.

### Decreasing feruloylation in grass biomass

The *AT* genes that modify GAX represent promising targets to improve the suitability of grass lignocellulosic biomass for biofuel production. Since FA on GAX is believed to be the main means by which polysaccharide is cross-linked to lignin, grass SCW FA amounts are therefore a key to recalcitrance. Suppression of the putative FAT-encoding *SvBAHD01*/*SvAT9* in the model grass *Setaria viridis* resulted in a ~40% increases in ease of digestion of cell wall polysaccharides into sugars in the modified plants compared with the wild type (de Souza et al., 2018). Similarly, suppression of the ortholog in sugarcane (*ScBAHD01*/*ScAT9*) improved the digestibility of sugarcane straw by approximately 20% after Organosolv pretreatment, compared to non-transformed plants (de Souza et al., 2019). These results are exciting because sugarcane (*Saccharum* spp.) covers vast areas of land (around 25 million ha worldwide), and its processing is already linked into infrastructure for producing bioethanol in many countries, especially in Brazil. Furthermore, sugarcane straw and bagasses are the main industrial residues after sugarcane processing (Menandro et al., 2017). Also, the Organosolv process involves the use of an organic liquid and water to partially hydrolyze lignin bonds and lignin-carbohydrate bonds, resulting in a solid residue consisting of mainly cellulose and some hemicellulose (Zhao et al., 2009). Thus, the biomass of suppressed *ScBAHD01*/*ScAT9* plants combined with Organosolv pretreatment is an interesting approach to be incorporated in the sugarcane industry for bioethanol production (de Souza et al., 2019). In addition, the reduction in FA-AX that often accompanies increases in *p*CA-AX due to altered expression of PATs in grasses, has been accompanied by a 10 to 40% increase in saccharification depending on the assay conditions. Thus, PAT enzymes like AT10 are also an attractive biotechnological target (Bartley et al., 2013; Li et al., 2018; Mota et al., 2021).

Recently, the world’s first CRISPR-edited sugarcane plants, the so-called Flex I and Flex II sugarcane, were reported (Brazilian Agricultural Research Corporation, 2021). Both Flex I and Flex II plants have CRISPR/Cas9-edited *AT* genes, and these sugarcane varieties presented higher cell wall digestibility and higher concentration of sucrose in plant tissues, respectively. The precise acyltransferase genes that were edited in these plants were not revealed, but both varieties have decreased levels of ferulate in the cell wall. Moreover, these CRISPR-edited plants were considered non-transgenic by the Brazilian National Technical Commission on Biosafety, representing an important step towards the use of this modified biomass by the bioethanol industry, as edited plants lacking foreign DNA can bypass the costly process of genetically modified-crop regulation.

### Zip-lignin (feruloyl lignin) and other lignin hydroxycinnamates

Feruloyl lignin, i.e., lignin containing feruloyl monolignol conjugates (ML-FAs), facilitates depolymerization of lignin polymers by industrial processes due to the introduction of mild base-labile ester bonds into the lignin polymer. Early work on this technology revealed that incorporation of synthetic coniferyl ferulate into lignin of cell cultures enhanced alkaline delignification and enzymatic hydrolysis (Grabber et al., 2008; Ralph, 2010). Wilkerson et al. (2014) then introduced the Chinese angelica feruloyl-monolignol transferase (AsFMT) to poplar to generate ML-FAs that were incorporated into lignin polymers. The resulting biomass presented improved saccharification after mild base pretreatment (Wilkerson et al., 2014). The generation of the “zip-lignins” can be achieved either through a linear linkage, by extending the polymer chain, or by crosslinking two lignin polymers, as demonstrated elsewhere (Ralph, 2010; Rencoret et al., 2013; Lu et al., 2015; Smith et al., 2015; Kaal et al., 2018). Both biophysical and chemical changes in cell wall accessibility have been observed due to the introduction of ML-FAs in poplar lignin (Shen et al., 2019). As discussed above, FMT activity has also been demonstrated for AT5s in grasses (Karlen et al., 2016). One way to boost the effect of this endogenous enzyme was demonstrated in maize by suppression of the first lignin specific biosynthetic enzyme, cinnamoyl-CoA reductase (CCR) resulting in an increase in the intercellular pool of feruloyl-CoA and in ML-FAs and an overall decrease in lignin content thereby enhancing the digestibility of stem rind tissue (Smith et al., 2017). Similarly, ectopic expression of *PMT* genes increased saccharification yields under some reaction pretreatment conditions both in Brachypodium (Petrik et al., 2014) and *via* heterologous expression in Arabidopsis (Sibout et al., 2016). The mechanism could be due to the tendency of *p*CA-acylated monolignols to end lignin polymerization and not be included within the lignin polymer, consistent with the greater alkali solubility of Arabidopsis lignin esterified with *p*CA (Sibout et al., 2016). These results show that ML-hydroxycinnamate conjugates are a promising means for engineering bioenergy crops and waste streams of mainstream crops by conferring low-cost lignin breakdown and separation for biorefining applications.

### Other biotechnological applications

Grains with increased feruloylated arabinoxylans are emerging as a potential multifunctional food and hydroxycinnamates themselves are being used as precursors for material applications. Some have reported that the presence of ferulic acid on the AX can contribute to antioxidant, anticancer and prebiotic properties (Srinivasan et al., 2007; Snelders et al., 2014). In addition, the cross-linking of FA-AX can form covalently linked gels with potential as drug delivery systems with anticancer or antioxidant properties (Mendez-Encinas et al., 2018). Recently, a study demonstrated the optimization of FA-AX isolation from wheat bran at a pilot scale using subcritical water extraction, demonstrating the feasibility of multifunctional FA-AX-based products for food and material applications in industrial scale (Rudjito et al., 2019). Furthermore, hydroxycinnamates in pure or mixed forms are themselves being used in higher value applications. For example, bacteria have been engineered to use HCAs to synthesize muconic acid (Johnson et al., 2016), a precursor of nylon. Techno-economic analyses indicate the value of engineering biomass to predominantly (>80%) produce only a single hydroxycinnamate for use as a high-value precursor (Karlen et al., 2020). Therefore, the manipulation of *AT* genes in different plant species can improve not only the production of biofuels but may also prove important for food and pharmaceutical applications.

## Outstanding questions

This review has highlighted some clear gaps in our knowledge that could be the focus of future research.

For plant science discovery:

- What is the acceptor molecule for ATs responsible for addition of FA and *p*CA to xylan and the pathway for their incorporation?
- Can experimental structural determination of AT enzymes and molecular docking studies explain their acceptor and donor substrate specificities?
- Evidence suggests that GAX-FA plays a key role in cross-linking between xylan chains and from xylan to lignin in grass cell walls; how is this cross-linking controlled?
- What are activities of the uncharacterized Mitchell subclade i and subclade ii ATs?
- What is the function (fitness advantage) of xylan *p*-coumarylation, lignin *p*-coumarylation and lignin feruloylation in commelinids?

For biotechnology applications it seems likely there are limits to manipulation of ATs before negative side effects occur:

- How much lignin FA and lignin *p*CA is too much?
- How much GAX-feruloylation is too little?

Addressing these questions will provide insight into the factors that have driven the evolution of grass cell wall properties, reveal molecular means to incorporate beneficial agronomic features associated with the hydroxycinnamates into food crop species, and potentially lead to the greater utilization of biomass and hydroxycinnamates themselves in the bio-economy.

## Author contributions

RM, CZ, WS, LB, and NC wrote the manuscript. NC and RM made the figures. LB, RM, WS, and NC revised the text and figures. All authors contributed to the article and approved the submitted version.
