# Corrigendum: Modification of plant cell walls with hydroxycinnamic acids by BAHD acyltransferases

**DOI:** 10.3389/fpls.2023.1163174

**Published:** 2023-02-21

**Authors:** Niharika Nonavinakere Chandrakanth, Chengcheng Zhang, Jackie Freeman, Wagner Rodrigo de Souza, Laura E. Bartley, Rowan A.C. Mitchell

**Affiliations:** ^1^ Institute of Biological Chemistry, Washington State University, Pullman, WA, United States; ^2^ Department of Microbiology and Plant Biology, University of Oklahoma, Norman, OK, United States; ^3^ Plant Sciences, Rothamsted Research, West Common, Harpenden, Hertfordshire, United Kingdom; ^4^ Center for Natural and Human Sciences, Federal University of ABC, Santo André, Brazil

**Keywords:** ferulic acid, para-coumaric acid, grasses, cell wall, xylan, lignin, plant biotechnology, bioenergy

In the published article, Feijao et al., 2022 was omitted from **Models for the mechanism of incorporation of HCAs into lignin and xylan**, *Mechanisms of hydroxycinnamoyl incorporation onto arabinoxylans*, Paragraph 3. The corrected sentence appears below:

“However more recent LC-MS analysis of sugar products released by mild acid treatment from the rice xax1 mutant suggests that XAX1 functions in the transfer of hydroxycinnamoyl-Araf to xylan, as all FA-Araf and pCA-Araf decorations of GAX were decreased in the mutant compared with the wild type (Feijao et al., 2022).”

In the published article, the reference for Fry (1986) was incorrectly written as “Fry, S.C. (1986). *In-vivo* formation of xyloglucan nonasaccharide: A possible biologically active cell-wall fragment. Planta 169(3), 443-453. doi: 10.1007/bf00392143”. The correct reference appears below:

“Fry, S.C. (1986). Cross-linking of matrix polymers in the growing cell walls of Angiosperms. Annual Review of Plant Physiology 37, 165-186”.

The authors apologize for these errors and state that this does not change the scientific conclusions of the article in any way. The original article has been updated.

